# Radiological progress report of curing scoliosis according to the fed method based on own material

**DOI:** 10.1186/1748-7161-9-S1-P14

**Published:** 2014-12-04

**Authors:** Zbigniew Śliwiński, Wojciech Kufel, Bartłomiej Halat, Beata Michalak, Danuta Śliwińska, Grzegorz Śliwiński

**Affiliations:** 1 Physiotherapy Center Zgorzelec, Zgorzelec, Poland; 2 Dresden University of Technology - Institute of Biomedical Engineering, Dresden, Germany

## Introduction

In the process of treating scoliosis X-ray is one of the objective methods of assessing the progress of therapy. In the assessment of scoliosis picture should cover the entire spine, hip bones with plates and hips, made standing in the AP and lateral projections. On the basis of a well-made images, you can specify the parameters of scoliosis (type of scoliosis, Risser test, the Cobb angle, the angle of rotation of the vertebrae, the index kifo - lordosis, etc.) which allows the selection of the proper physiotherapy and assessment of treatment effects.

## Materials and methods

We evaluated a group of 70 children diagnosed with idiopathic scoliosis in age from 7 to 18 years residing in the treatment by the Fed at the Centre for Rehabilitation in Zgorzelec. The children remained in the two monthly turnusach apart semester. During the stay twice a day participated in therapy by the Fed. Analysis and evaluation of X-ray were performed before treatment and at the end of the half-year stage. With images were evaluated Cobb angle, vertebral rotation by raimondii test Risser, type of scoliosis by King-Moe.

## Results

The results have been developed in the form of tables and charts, broken down by the scoliosis to 20 °, 30 °, 40 ° and above 40 °.

## Conclusions

Comparison of X-ray images is one of objective assessment in the treatment of scoliosis. The results presented in the study are the evaluation of the effectiveness of the method the Fed.

